# Therapy-Induced Cellular Senescence: Potentiating Tumor Elimination or Driving Cancer Resistance and Recurrence?

**DOI:** 10.3390/cells13151281

**Published:** 2024-07-30

**Authors:** Yue Liu, Isabelle Lomeli, Stephen J. Kron

**Affiliations:** Ludwig Center for Metastasis Research and Department of Molecular Genetics and Cell Biology, The University of Chicago, Chicago, IL 60637, USA

**Keywords:** senescence, SASP (senescence-associated secretory phenotype), immune surveillance, immunosuppression, senolytics, tumor microenvironment, cancer therapy, therapy resistance

## Abstract

Cellular senescence has been increasingly recognized as a hallmark of cancer, reflecting its association with aging and inflammation, its role as a response to deregulated proliferation and oncogenic stress, and its induction by cancer therapies. While therapy-induced senescence (TIS) has been linked to resistance, recurrence, metastasis, and normal tissue toxicity, TIS also has the potential to enhance therapy response and stimulate anti-tumor immunity. In this review, we examine the Jekyll and Hyde nature of senescent cells (SnCs), focusing on how their persistence while expressing the senescence-associated secretory phenotype (SASP) modulates the tumor microenvironment through autocrine and paracrine mechanisms. Through the SASP, SnCs can mediate both resistance and response to cancer therapies. To fulfill the unmet potential of cancer immunotherapy, we consider how SnCs may influence tumor inflammation and serve as an antigen source to potentiate anti-tumor immune response. This new perspective suggests treatment approaches based on TIS to enhance immune checkpoint blockade. Finally, we describe strategies for mitigating the detrimental effects of senescence, such as modulating the SASP or targeting SnC persistence, which may enhance the overall benefits of cancer treatment.

## 1. Introduction 

Cellular senescence was first described as an in vitro phenomenon known as the Hayflick limit [1], recognizing the loss of proliferative potential during serial passage as a form of cellular aging now known as replicative senescence (RS). It took several decades to establish that RS is linked to telomere shortening in human tissues and that senescent cells (SnCs) accumulate during aging [2,3]. These discoveries dispelled the argument that cellular senescence was merely an artifact of cell culture. Along with telomere erosion during proliferation, senescence can be triggered by replication fork collapse, oxidative damage, oncogenic stress, and cancer therapies, all of which may lead to irreparable DNA damage [4]. Despite entering a prolonged growth arrest, SnCs remain metabolically active. SnCs typically express a characteristic range of proteins that either decorate the surface or are released into their microenvironment known as the senescence-associated secretory phenotype (SASP) [5]. The release of cytokines and other SASP factors as well as concomitant metabolic changes that produce other signaling mediators can alter cellular behaviors in an autocrine and/or paracrine manner [6,7,8,9,10]. A striking effect is the ability of SnCs to induce bystander senescence in neighboring cells [11], propagating senescence through tissues and providing a cellular basis for progressive organ dysfunction and organismal aging.

Beyond its connection to aging, senescence has been recognized as a hallmark of cancer [6]. Whether senescence is beneficial or detrimental to cancer initiation, progression, and/or treatment has remained controversial over the past few decades. On one hand, the induction of senescence can serve as a barrier against malignant transformation and excessive hyperproliferation due to reduced proliferative capacity [12,13]. On the other, SnC accumulation may act as a driver of cancer progression and therapy resistance, primarily mediated by inflammatory factors in the SASP [14]. Persistent senescence has been associated with promoting malignant transformation, accelerating tumor growth, inducing cancer stemness, facilitating distant metastasis, maintaining chronic inflammation, and dampening the anti-tumor immune response [15,16,17,18]. Confirming these deleterious effects, genetic elimination of SnCs was shown to delay spontaneous tumorigenesis and decrease cancer-related mortality [19,20,21,22]. Senolytic agents, drugs that selectively eliminate SnCs [19], have also demonstrated significant potential in improving cancer therapies [10,19,23,24,25]. Nonetheless, our studies have identified opposing, apparently beneficial, effects of TIS, where SnCs may serve as a vaccine to drive an adaptive immune response to inhibit tumor growth and boost radiation therapy [26]. This work is built on pioneering studies revealing innate and adaptive immune recognition of SnCs leading to their elimination and tumor suppression [27,28,29], often described as senescence surveillance. Over the past decade, a growing literature has appeared that further defines roles for host immunity in mediating the anti-tumor effects of SnCs, as reflected in recent reviews [30,31,32]. Multiple studies have implicated the upregulation of inflammatory mediators including DAMPs, chemotactic factors and other cytokines, and antigen presentation machinery in the activation of both innate and adaptive immune responses, not only driving SnC elimination via immune surveillance but also potentiating broader immune responses [30,31,32]. Such findings highlight the positive aspects of TIS, extending beyond growth suppression to significantly enhance anti-tumor immunity. A factor underlying the apparent inconsistency may be that SnCs, including those formed by cancer therapies, can express immune checkpoint ligands that allow SnCs to evade surveillance and protect their microenvironment [33,34,35,36,37]. Thereby, some benefits of immune checkpoint blockade (ICB) therapy in combination with genotoxic or targeted therapies may be mediated by overcoming immunosuppression driven by TIS and restoring immune surveillance.

This review examines cellular senescence in the context of cancer, highlighting the diverse roles of SnCs in the tumor microenvironment (TME) and arguing for a broad view of senescence and its functions. We will discuss how the interaction between SnCs and the immune system can lead to either beneficial or detrimental outcomes depending on the specific features of SnCs, particularly the SASP. We will then review SASP modulation and SnC elimination via senolytics. Such approaches may limit the adverse effects of senescence while amplifying its beneficial impact, which ultimately presents an alternative strategy to improve cancer therapies.

## 2. Senescence in the Context of Cancer

Senescence is a normal fate for cells in diverse physiological contexts, such as embryonic development [38,39], wound healing [40,41], normal aging [42], viral infection [43], and other stress-related conditions. In cancer, SnCs can originate from different sources and are detected in premalignant lesions as well as within the TME during different phases of cancer initiation, progression, and therapy response [44,45]. These non-proliferative tumor cells can be triggered by diverse stimuli, including reactive oxygen species (ROS), DNA damage, imbalanced cellular signaling networks, and epigenetic alterations [10,24].

One of the core pathways linking DNA damage to senescence is the tumor suppressor and transcription factor p53, whose activation drives cell cycle arrest via the expression of the cyclin-dependent kinase inhibitor (CDKi) p21^CIP^ (also known as p21^Waf1^ or p21) and potentially a second CDKi, p16^INK4A^ (also known as p16) [46,47]. The pro-apoptotic effects of p53 appear to be offset in SnCs by other factors that maintain cell survival, such as Bcl-2 family proteins [48].

### 2.1. Oncogene-Induced Senescence (OIS)

Oncogene activation is a potent inducer of cellular senescence. Initial studies demonstrated that cultured primary mammalian cells with oncogenic Ras overexpression undergo senescence in a p53-dependent manner in vitro [49]. Subsequent work in the mammary gland showed that deregulated Ras activity induces senescence in vivo [50]. Other oncogenes such as Mos, Cdc6, and Cyclin E have also been described as senescence inducers, reinforcing the notion that OIS provides a barrier to malignant progression [51]. The mechanisms of OIS are notably linked to DNA damage signaling [51,52]. Ras activation has been shown to trigger DNA hyper-replication, leading to replication fork stalling and collapse [53] and telomere attrition [54]. Such events can elicit the DNA damage response (DDR) and senescence induction. Oncogene activation is also associated with increased ROS that may initially drive cell proliferation, but can ultimately cause DNA damage, cell cycle arrest, and senescence [55,56]. Along with oncogene activation, tumor suppressor inactivation can also induce cellular senescence [57,58]. One study found that loss of the tumor suppressor PTEN induces DNA damage, leading to senescence in mouse prostate epithelial cells [57]. Other studies alternatively suggest that PTEN loss can cause senescence independently of DNA damage through increased p53 stabilization and enhanced transcription of p53 targets such as p21^CIP^ [58].

### 2.2. Therapy-Induced Senescence (TIS)

Entering a persistent, senescence-like arrest rather than undergoing cell death in response to genotoxic stress from radiation or chemotherapies is described as therapy-induced senescence (TIS) [15,59,60]. As an initiating event, DNA damaging agents used in cancer treatment generate DNA single- or double-strand breaks at telomeres [61] and in other chromosomal regions [62], activating a DDR via ATM, DNA-PKcs, ATR, CHK1, and CHK2 and/or other signaling kinases that converge at the tumor suppressor p53, resulting in cell cycle arrest [63]. While proliferation may resume when DNA damage is repaired, irreparable DNA damage induces a prolonged DDR and extended growth arrest that can progress into senescence [64]. Inhibiting DDR kinases such as ATM or their target p53 may allow cells to bypass senescence or even permit SnCs to re-enter the cell cycle [65,66].

Although activation of the p53-dependent G1 cell cycle checkpoint is often described as critical in TIS, p53-negative cancer cells can also be driven into senescence. Similarly, activation of the DDR is not required. Many targeted agents that do not induce genotoxic stress can promote TIS [67]. Consistent with studies that showed enforcing G1 arrest whether via blocking CDK2 activity by overexpressing p21^CIP1^ or targeting CDK4/6 by overexpressing p16^INK4A^ is sufficient to induce senescence, CDK4/6 inhibitors such as palbociclib or abemaciclib can each lead to senescence [68].

Cells can also enter senescence after perturbation of G2 or M phase [69]. The transient G2 arrest due to CHK1/2-mediated inhibition of Cdc25 and failure to activate CDK1 can become permanent via expression of p21^CIP1^, which mediates premature activation of APC/C^Cdh1^ and degradation of cyclin B1 [69,70,71]. After treatment with spindle poisons or other agents that disrupt mitosis, surviving cells may enter senescence without DNA damage [72]. Blocking cytokinesis by inhibiting Aurora kinases or Polo-like kinase 1 results in mitotic slippage, leading to binucleate G1 cells that progress to senescence [73,74].

Targeting chromatin modifications with epigenetic drugs, such as the histone deacetylase inhibitors sodium dibutyrate (SDB) and trichostatin A (TSA) [75], can induce senescence via a mitotic slippage-mediated pathway [76]. Therapy-induced DNA damage may similarly result in mitotic slippage following prolonged checkpoint arrest [77]. Cells responding to ROS exposure or telomere erosion also display mitotic defects and cytokinesis failure followed by cell cycle arrest and onset of senescence [78,79].

## 3. Hallmarks of Cellular Senescence

**Figure 1:**
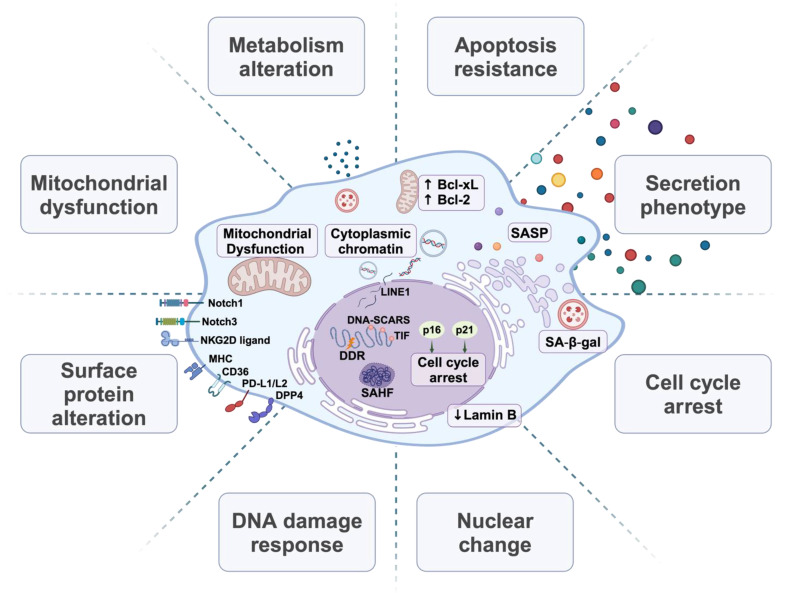
**Hallmarks of cellular senescence.** Cellular senescence is characterized by an exit from proliferation and persistent cell cycle arrest, accompanied by a host of cellular changes. In culture, SnCs display an enlarged and flattened morphology, altered nuclear shape, and prominent intracellular vesicles, which can be identified through phase contrast or brightfield microscopy. The increased vesicular content likely underlies the activation of GLB1 lysosomal β-galactosidase, a biochemical marker known as senescence-associated β-galactosidase (SA-β-gal), detectable with X-Gal or other chromogenic and fluorogenic β-galactosidase reporters. Other features include a chronically active DNA damage response (DDR), characterized by persistent nuclear foci of phosphorylated histone H2AX. These foci may form DNA segments with chromatin alterations reinforcing senescence (DNA-SCARS) or telomere dysfunction-induced foci (TIF) when associated with promyelocytic leukemia protein (PML) nuclear bodies. Additional nuclear changes in SnCs include decreased lamins, chromatin remodeling, and senescence-associated heterochromatin foci (SAHF). SnC persistence reflects resistance to apoptosis through the overexpression of anti-apoptotic proteins such as the Bcl-2 family members. Another hallmark of senescence is the accumulation of dysfunctional mitochondria, contributing to increased levels of reactive oxygen species (ROS) and oxidative damage. SnCs also display altered surface protein expression. Despite growth arrest, SnCs remain metabolically active and experience significant metabolic changes. A key feature of SnCs is the senescence-associated secretory phenotype (SASP), characterized by the release of a wide range of bioactive molecules and extracellular vesicles. The accumulation of single and double-stranded cytosolic DNA and chromatin fragments, including those from retrotransposon reverse transcription via LINE1, chromosomal DNA damage processing, dysfunctional mitochondria, and micronuclei, can contribute to SASP through the cGAS-STING pathway. MHC, major histocompatibility complex; DPP4, dipeptidyl peptidase 4.

SnCs typically display a characteristically enlarged and flattened cell morphology, altered metabolism, and activation of the lysosomal enzyme GLB1 as detected via senescence-associated beta-galactosidase (SA-β-Gal) [80], and changes in gene expression and signaling (Figure 1). Many hallmarks of cellular senescence have been used individually or in combination to identify SnCs in clinical studies [81]. However, some features of SnCs are particularly heterogeneous, likely reflecting the variety of senescence-inducing mechanisms [8]. Additionally, features often described as characteristic of senescence, including SA-β-Gal and the SASP, substantially vary and are observed in cells that do not otherwise display senescence phenotypes [8]. These uncertainties have prompted the development of SnC detection methods both in vitro and in-tissue [82,83,84], but also create additional challenges in linking senescence to cancer.

### 3.1. The DNA Damage Response

SnCs often display a persistent DDR associated with constitutive staining for nuclear foci of phosphorylated histone H2AX (γH2AX), p53-binding protein (53BP1), or phosphorylated ATM [64,85]. The DDR in these cells can be initiated by cell-intrinsic factors, including telomeric damage, DNA replication errors, or replication fork collapse [52,86]. Genotoxic agents that induce single- or double-strand breaks in chromosomal DNA, such as radiation and chemotherapeutic drugs, can also serve as potent DDR activators [87,88]. Prolonged DNA damage foci have been detected in the undamaged chromatin of some SnCs that may be attributed to hyperactive ATM activity [77]. A critical component of the DDR signaling cascade is p53, which undergoes activating phosphorylation by the DDR-associated kinases ATM, CHK1, and CHK2, reducing affinity for the MDM2 E3 ubiquitin ligase and stabilizing p53 levels [87]. The transcriptional activity of p53 then drives the expression of genes that initiate and maintain the senescence phenotype [66]. Additionally, CHK1-mediated phosphorylation inhibits CDC25, blocking the G2/M transition to induce G2 arrest [89]. The DDR kinase cascade also influences other signaling pathways, including NF-κB, STING, MAPK, and STAT, which likely contributes to the characteristics of senescence beyond cell cycle arrest [90,91].

### 3.2. CDK Inhibitors and Cell Cycle Arrest

A DDR-related hallmark of SnCs is the upregulation of CDK inhibitors [92]. In particular, the expression of p16^INK4A^ (encoded by *CDKN2A*) remains low in healthy, young tissues, but increases under senescence-inducing stresses and serves as a valuable senescence marker [42]. p16^INK4A^ selectively blocks the activity of cyclin D-dependent kinases CDK4 and CDK6 and facilitates Rb-associated cell cycle arrest [93,94]. Demonstrated in INK-ATTAC and p16-3MR mouse models, the targeted removal of p16^INK4A^-positive cells may improve both health span and lifespan [22,41,95,96].

The *CDKN2A* gene also encodes p14^ARF^ in human or p19^ARF^ in mouse, an alternate reading frame protein that shares p16^INK4A^ exons 2 and 3 [97]. ARF modulates the cell cycle through interaction with MDM2 to enhance p53-dependent gene transcription [98,99]. Another INK4 family member, p15^INK4B^ (encoded by *CDKN2C*), may compensate in the absence of p16^INK4A^ [100]. For example, Akt-mediated p15^INK4B^ expression is required for senescence in response to androgen stimulation in prostate cancer cells [101].

The expression of p21^CIP^, encoded by the p53 target gene *CDKN1A*, also increases upon senescence-inducing stimuli [102]. Although capable of inhibiting multiple cyclin/CDK complexes, p21^CIP^ predominantly induces G1 cell cycle arrest via blocking CDK2 [103]. Beyond p53, p21^CIP^ induction can also result from p53-independent stress signals, including TGF-β, Rb, and integrin, which facilitate Sp1/Sp3 transcription factor recruitment to the p21^CIP^ promoter and increase p21^CIP^ expression [104]. Unlike the consistent upregulation of p16^INK4A^, p21^CIP^ levels vary in senescence [105], which limits the value of p21^CIP^ as a senescence marker.

### 3.3. Chromatin and Nuclear Changes

SnCs undergo significant changes in chromosome structures and epigenetic modifications. Some notable nuclear substructures reflecting persistent DDR in SnCs are DNA-SCARS (DNA segments with chromatin alterations reinforcing senescence). These structures contain activated DDR proteins, such as p53 and CHK2, and are associated with promyelocytic leukemia protein (PML) nuclear bodies, which are dynamic, membraneless structures triggered by cellular stress [106]. PML nuclear bodies can also harbor damaged telomeres and form telomere dysfunction-induced foci (TIF) in replicative SnCs [106]. Additionally, senescence-associated heterochromatic foci (SAHF), marked by large nucleoli and punctate DNA foci visible after DAPI staining, represent a distinct heterochromatic structure that correlates with the stable repression of E2F-targeted genes [107]. The development of SAHF and associated chromatin structures depends on the histone chaperone proteins ASF1A and HIRA [108], which are influenced by signaling pathways such as Rb and the NOTCH-HMGA1 axis [107,109]. Notably, SAHF formation is observed in human cells experiencing accelerated senescence in an ATR-dependent manner and is less associated with RS or mouse cells [92,110].

Another feature of SnCs is the decrease in nuclear inner membrane protein Lamin B1 [111,112]. This reduction is linked to large-scale chromatin remodeling, including SAHF formation [113]. The decline in Lamin B1 also destabilizes the nuclear envelope [114]. This may facilitate the release of cytoplasmic chromatin fragments (CCFs) that trigger the cGMP-AMP synthase stimulator of interferon genes (cGAS-STING) pathway, amplifying the SASP and subsequent inflammation [115].

Epigenetic remodeling of senescence-associated gene expression is also a hallmark of SnCs. Reduced Lamin B may lead to redistribution of the active mark H3K4me3 and repressive mark H3K27me3 [116]. The silencing mark H3K9me2 may be reduced in SnCs due to DDR-induced proteasomal degradation of methyltransferases G9a and GLP [117]. Activation of histone acetyltransferase p300 and subsequent acetylation may support senescence-specific gene expression [118], likely through engagement of histone acetylation reader protein BRD4 in activating super-enhancers [119]. Epigenetic changes also occur at DNA repetitive elements, such as transposable element (TE) sequences [120,121]. In SnCs, a decrease in histone trimethylation (H3K9me3 and H3K27me3) has been observed in the region of the class I TE long-interspersed element-1 (LINE1), leading to its increased transcription and activation [120]. In turn, upregulated LINE1 RNA suppresses histone-lysine N-methyltransferase SUV39H1, contributing to heterochromatin loss and other senescent phenotypes [122]. Accumulated LINE1 cDNA also triggers type-I interferon production in a cGAS-dependent mechanism [120].

### 3.4. Resistance to Apoptosis

While cellular senescence and apoptosis can be triggered by similar stimuli [20], senescent human fibroblasts display resistance to p53-dependent apoptosis [123]. One factor may be persistent expression of the anti-apoptotic protein Bcl-2, providing a direct mechanism for evading apoptosis [124]. The Bcl-2 gene in SnCs shows enhanced H4K16 acetylation but diminished H4K20Me3, which suggests active transcription, whereas BAX expression is repressed by the reverse modification pattern [125]. Other Bcl-2 family proteins, such as Bcl-w and Bcl-xl, are upregulated in both RS and OIS [25,126]. Additionally, p21^CIP^ may inhibit NF-κB and subsequent JNK-cascade-dependent cell death in SnCs [127]. FOXO4 is another protein that enhances the survival of SnCs via blocking p53-induced apoptosis [128]. SnCs also display increased levels of other pro-survival proteins, including HSP90, Ephrins (EFNB1 or 3), PI3Kδ, and plasminogen-activator inhibitor-2 (PAI-2) [129,130]. Senolytics can induce apoptosis and selectively eliminate SnCs by targeting these anti-apoptosis mechanisms [25,129,130,131].

### 3.5. Dysfunctional Mitochondria

Mitochondrial dysfunction is recognized as a hallmark of cellular senescence and referred to as senescence-associated mitochondrial dysfunction (SAMD) [132,133]. SnCs display downregulation of fission protein 1 (Fis1) and dynamin-related protein 1 (Drp1), leading to an imbalance in mitochondrial fission and fusion. This leads to the formation of hyper-fused mitochondria with altered morphology and function [134]. The accumulation of dysfunctional mitochondria is further compounded by reduced mitophagy that delays mitochondrial turnover [133]. Mitochondrial dysfunction may be a direct cause of senescence, considering mitochondrial depletion can prevent senescence in vitro and in vivo [135].

Increased mitochondrial mass and/or persistence correlates with higher oxygen consumption in TIS and OIS [136,137]. Impaired mitochondria are associated with increased ROS that can induce DDR and reinforce senescence [138,139]. Depending on how hypoxia or hyperoxia influences ROS, the impact of oxygen tension on senescence induction can vary [140]. Both impairing mitochondrial respiration complexes and enhancing respiration via pyruvate dehydrogenase can increase redox stress and senescence [141,142]. Redox stress may also arise from accumulated labile iron that may further contribute to the SASP in SnCs [143]. The loss of antioxidant enzymes such as superoxide dismutase (SOD) can also drive senescence [141].

### 3.6. Deregulated Metabolism

**Figure 2:**
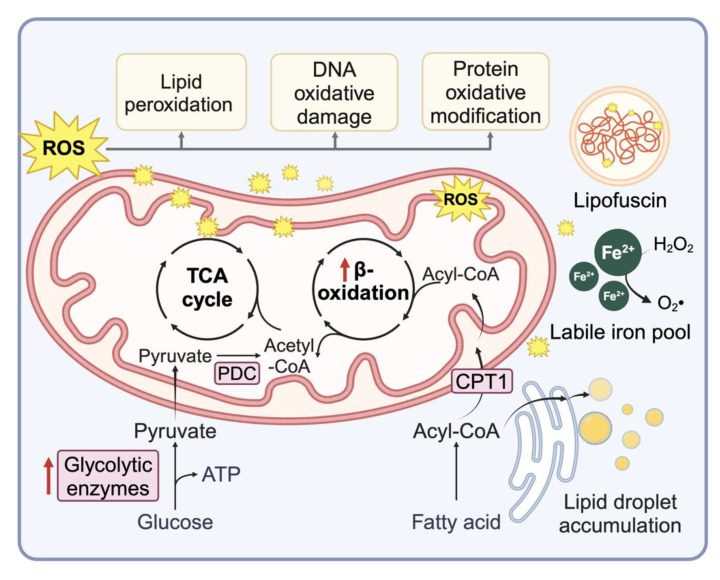
**Metabolic changes in senescent cells.** SnCs can display a Warburg effect-like pattern by continuing glycolysis regardless of oxygen availability. This shift is often accompanied by the upregulation of glycolytic enzymes. SnCs may also exhibit enhanced beta-oxidation and oxygen consumption. Lipid metabolism shifts toward increased fatty acid synthesis, along with increased lipid uptake, resulting in lipid droplet accumulation. Overall, SnC metabolic changes can contribute to elevated ROS levels. As ROS levels rise, oxidative damage occurs across various cellular components, causing depletion of antioxidants, lipid peroxidation, DNA damage, and protein carbonylation. Labile iron pools in SnCs can exacerbate oxidative damage by facilitating the conversion of relatively mild oxidants into highly reactive free radicals via the Fenton reaction. Furthermore, oxidative stress compromises the lysosomal and proteasomal systems. These changes contribute to the formation of lipofuscin, a complex aggregate of highly cross-linked non-degradable proteins, carbohydrates, lipids, and transition metals.

Lipid accumulation, lipid signaling, and deregulated lipid metabolism have long been linked to cellular senescence (Figure 2). Nearly three decades ago, Obeid and colleagues found that ceramide levels increase as cells enter RS, and feeding cells ceramide was sufficient to induce accelerated senescence in otherwise proliferative fibroblasts [144]. The pro-senescent effects of ceramide may be linked to mitochondrial dysfunction, altered mitophagy, and ROS production [145,146]. Cholesterol biosynthetic pathways appear to modulate senescence by similar mechanisms, albeit more indirectly [147,148]. Exposing proliferative cells to free fatty acids can also induce senescence by increasing oxidative stress [149,150].

Together with sphingolipids, multiple lipid metabolic pathways are altered in SnCs [151,152,153]. Our previous studies found that activation of diverse lipid metabolism pathways and increased lipid uptake led to the accumulation of lipid droplets (LDs) in DNA-damage-induced SnCs [150]. An increase in lipid oxidative damage also leads to the accumulation of reactive aldehyde and the upregulation of responsive proteins, such as aldehyde dehydrogenases and lysosomal palmitoyl-protein thioesterases [150]. Lipid peroxidation, where unsaturated lipids are oxidized by a free-radical-driven chain reaction that yields lipid-derived electrophiles, is a common feature in multiple forms of senescence. Lipid peroxidation-derived aldehydes such as 4-hydroxynonenal (HNE) accumulate and form protein adducts in SnCs [150]. They can induce senescence on their own and have been implicated in mediating bystander senescence in tissues [11,150]. Mitochondria may serve as key modulators, as they are sites of lipid peroxidation via cardiolipin oxidation and targets for lipid aldehyde-adduction to proteins, nucleic acids, or other lipids [154]. Senescent T cells also demonstrate increased fatty acid oxidation and synthesis, especially in cholesterol esters, contributing to LD accumulation via a cPLA2α-dependent pathway [155]. Inhibition or knockdown of cPLA2α in T cells reduces LD accumulation, prevents senescence induction, and promotes effector T cell functions and anti-tumor immunity [155].

Intracellular accumulation of lipofuscin, a pigmented aggregate of nondegradable cellular debris, proteins, lipids, and transition metals, has long been associated with tissue aging and neurodegeneration and may serve as a biomarker for cellular senescence [156,157]. Lipofuscin forms through the crosslinking of proteins by lipid peroxidation-derived electrophiles [158]. In SnCs, lipofuscin displays autofluorescence between 450 and 490 nm and co-localizes with SA-β-Gal [156,159]. Staining for lipofuscin with Sudan Black B or its analogs, such as GLP13 and GLP16, has been applied to detect senescence in vitro and in vivo [156,160,161,162].

Cancer cells often fail to display a Pasteur effect and continue to consume glucose in the presence of oxygen, a phenomenon known as the Warburg effect [163]. Aerobic glycolysis has also been observed in SnCs, likely due to mitochondrial dysfunction. In radiation-induced senescent human colon cancer cells, for example, glycolysis was reported as the primary energy source (Figure 2) [164]. While enhanced glucose metabolism can help cancer cells bypass OIS, a hyperglycemic state may accelerate senescence [165]. 2-deoxy-D-glucose (2DG) induces apoptosis more effectively in cyclophosphamide-induced senescent lymphoma cells than in non-SnCs, highlighting a potential senescence-specific vulnerability to glycolysis inhibitors [166]. Mitochondrial dysfunction affects other metabolic pathways as well. For instance, an altered NAD^+^/NADH ratio in SnCs influences the SASP [167,168]. NAD^+^-dependent protein SIRT1 is reduced through autophagosome–lysosome degradation during senescence [169].

### 3.7. Alterations in Surface Proteins

SnCs often display altered surface proteins [170]. Proteomics analysis of plasma membrane-associated proteins has identified multiple surface proteins preferentially expressed in SnCs that are driven by ectopic expression of p21^CIP^ or p16^INK4A^ compared to proliferating cells [171]. The major histocompatibility complex class I (MHC I) molecules or their component β2 microglobulin (β2M) may be upregulated in stress-induced SnCs [172]. In addition to MHC I, SnCs exhibit other surface alterations that can directly regulate the immune response. These include the upregulation of immune-active molecules, such as MHC II and NKG2D ligands (e.g., ULBP2 and MICA) [173,174], as well as immune-suppressive molecules including the non-classical MHC class I molecule HLA-E [175], B7 protein family members PD-L1 and PD-L2 [34,35,36,37], and the “Don’t Eat Me” signals CD47 and CD24 [176]. The roles of these molecules in anti-tumor immunity will be discussed in Section 4.4 and Section 5.4. Some of these surface proteins, such as NKG2D ligands, can be cleaved from the surface of SnCs by matrix metalloproteinases (MMPs) to evade immune surveillance [177].

Similar proteomic approaches have identified Notch1 as elevated in H-Ras^12V^-induced SnCs, contributing to a TGF-β-rich secretome [178]. Another Notch family member, Notch3, is also increased in TIS [179]. Proteomics analysis of RS has revealed an increased expression of dipeptidyl peptidase 4 (DPP4, also named CD26), which may provide a biomarker for senescence detection [180]. Inhibiting or silencing DPP4 expression can decrease SASP production and senescence progression [181,182]. Transcriptional analysis has also uncovered changes in surface proteins in SnCs, such as CD36, which is required for driving NF-κB dependent pro-inflammatory cytokine production and cell cycle arrest [183]. The urokinase-type plasminogen activator receptor (uPAR) was found to be upregulated in TIS, OIS, and RS [21]. Consequently, Chimeric Antigen Receptor T (CAR-T) cells that target uPAR can selectively eliminate SnCs both in vitro and in vivo [21]. Besides proteins alone, the N- and O-glycan modifications such as sialylation may be decreased on cell surface proteins upon senescence induction [184,185].

### 3.8. Regulation of the SASP

Cells undergoing senescence typically adopt an altered pattern of metabolite release, protein secretion, and vesicle release that mediate autocrine, juxtacrine, paracrine, and systemic effects. These are typically described as features of the senescence-associated secretory phenotype (SASP), a mix of pro-inflammatory factors including cytokines, chemokines, growth factors, proteases, lipids, extracellular components, MMPs, nucleic acids, and extracellular vesicles [186]. Along with proteins released by conventional secretory pathways, the SASP also includes macromolecules released via passive leakage through membrane pores and defects, exocytosis of vesicles, and shedding of transmembrane protein ectodomains that are cleaved by enzymes such as disintegrin and ADAM17 [187,188].

Previous reviews have summarized the extensive signaling pathways implicated in SASP regulation [7,8,189,190,191,192,193]. SnCs induced without DNA damage through ectopic expression of p16^INK4A^ or p21^CIP^ typically do not promote inflammation, suggesting an association between the proinflammatory SASP and stress responses [194]. Depleting DDR proteins like ATM, NBS1, or CHK2 [90,106,195], or inhibiting stress-induced p38 mitogen-activated protein kinases (MAPKs) [196] can diminish inflammatory SASP secretion. These stress signals predominantly activate NF-κB and C/EBP-β and drive the production of proinflammatory cytokines such as IL-1α, IL-6, and IL-8 [19,197,198,199,200]. SASP regulation also involves autocrine feedback loops. IL-6, for example, can reinforce senescence and promote a self-amplifying secretion network [201]. Surface-bound IL-1α can also initiate a positive loop that enhances IL-6 and IL-8 secretion [202]. IL-1 receptor signaling, on the other hand, elevates microRNAs 146a and 146b, creating negative feedback that limits SASP expression [203]. Notch1, often upregulated in SnCs, modulates SASP composition by negatively regulating the proinflammatory transcription factor C/EBP-β, which shifts the SASP toward a TGF-β-rich secretome [178,204]. A positive feedback loop between TGF-β and Notch further contributes to G1 cell cycle arrest and subsequent senescence [205,206]. TGF-β may also promote paracrine senescence by increasing ROS and DDR via NADPH oxidase 4 (Nox4) and activating p21^CIP^ signals [39,207].

Mammalian TOR (mTOR) signaling, a key regulator of lifespan and aging [208], also plays a critical role in SASP production [209,210]. During senescence, mTOR activity leads to 4EBP1 phosphorylation and MAPKAPK2 translation. This drives ZFP36L1 phosphorylation and prevents the degradation of SASP-related transcripts [211]. Inhibiting mTOR using drugs like rapamycin can effectively reduce inflammatory cytokine expression by suppressing IL-1α translation and diminishing NF-κB activity [212]. Such findings identify mTOR inhibitors as potential SASP suppressors that can alleviate related pathologies [213].

Recent studies have identified the cGAS-STING pathway as another central regulator of the SASP. In SnCs, cGAS senses single- and double-stranded cytosolic DNA and chromatin fragments, including those derived from retrotransposon reverse transcription, chromosomal DNA damage processing, dysfunctional mitochondria, and micronuclei. It then generates the secondary messenger cGAMP to activate the adaptor protein STING [120,214,215]. STING recruits TBK1 and IκB kinase, activating IFN regulatory factor 3 (IRF3) [216] and NF-κB [217], leading to the secretion of type I interferons and inflammatory cytokines [218]. STING activation can also promote the expression of Toll-like receptor 2 (TLR2) in OIS, further driving SASP via p38-MAPK activity [219]. Inhibiting STING signaling reduces SASP production and its associated functions in SnCs [215,220]. Deficiency of cGAS alters senescence primarily because of changes in the SASP, as well as altered oxidative stress response [214,218]. MRE11, a protein that contributes to micronuclei formation, is involved in cGAS activation and subsequent SASP production [221]. Conversely, three prime repair exonuclease 1 (TREX1) prevents cGAS-STING pathway activation by degrading cytosolic DNA [221,222]. TREX1 inhibition alone is sufficient for inducing replication stress, DNA damage, and senescence [221,222,223].

The inflammasome pathway also plays a role in SASP secretion. Gasdermin D (GSDMD), a downstream effector of inflammasome signaling, forms pores that mediate release of SASP factors [224]. Knocking down NLRP1, a major component of the inflammasome upregulated by cGAS, significantly attenuates SASP in IR-induced SnCs [224]. While NLRP3 appears to be less relevant in TIS, activation of the NLRP3 cascade acts as a driver of senescence and SASP in the aged mouse aorta [225].

Metabolic processes may also affect the SASP. During OIS, high mobility group A (HMGA) proteins promote the expression of nicotinamide phosphoribosyl transferase (NAMPT), the primary enzyme for NAD^+^ biosynthesis. This leads to a high NAD^+^/NADH ratio that activates p38-MAPK and inhibits AMPK, thereby increasing the expression of proinflammatory SASP. Supplementing senescent fibroblasts with nicotinamide mononucleotides (NMNs) further elevates the NAD^+^/NADH ratio and encourages the secretion of inflammatory cytokines [168]. Raising NAD^+^ levels in mouse brains through nicotinamide riboside (NR) administration conversely reduces inflammation by suppressing cGAS-STING signaling [226], emphasizing the context-dependent effects of NAD metabolism on SASP.

SASP regulation is further influenced by epigenetic modifications [227]. The diminished H3K9Me2 at SASP gene promoters contributes to IL-6 and IL-8 expression [117]. Inhibition of histone deacetylase (HDAC) can activate a proinflammatory SASP in the absence of DNA damage, likely reflecting chromatin hyperacetylation [228]. Downregulating the NAD-dependent deacetylase sirtuin-1 (SIRT1) during senescence allows increased H3K9 and H4K16 acetylation at SASP promoters [229]. SnCs show elevated levels of the histone variant macroH2A1, which stimulates SASP gene expression via a positive feedback loop [230]. Other epigenetic regulators, such as BRD4, HMGB1, HMGB2, GATA4, and MLL1, have also been identified as SASP modulators [227,231].

## 4. Senescence as an Ally in Cancer Treatment

**Figure 3:**
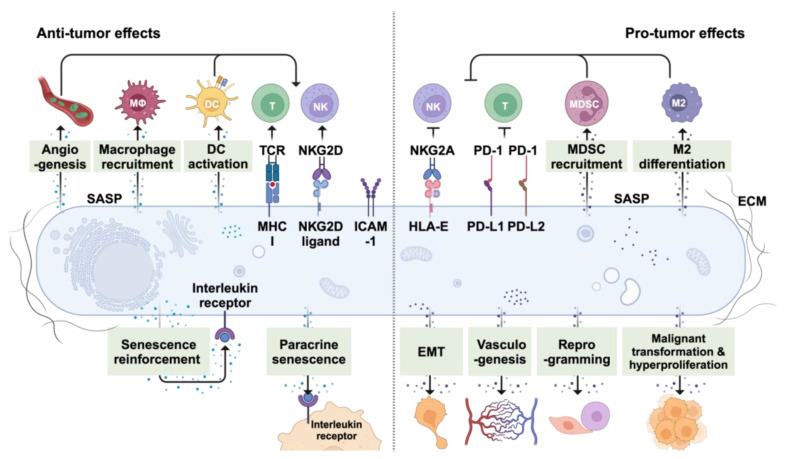
**Positive and negative effects of cellular senescence in the tumor microenvironment:** (**Left**) The anti-tumor effects of cellular senescence. SnCs contribute to anti-tumor defenses by facilitating the recruitment and activation of immune cells such as phagocytes (e.g., Mφ), dendritic cells (DCs), T cells, and natural killer (NK) cells. This is achieved through SASP factors and surface proteins, including class I MHC, NKG2D ligands, and ICAM-1. Ds activation by SnCs provides an additional mechanism for cytotoxic lymphocyte-mediated tumor control. Senescence can also promote angiogenesis, which contributes to the mobilization of immune cells to the tumor site. Beyond immune-mediated actions, SnCs can maintain and spread the senescent state through autocrine and paracrine mechanisms that suppress cell proliferation, with interleukin signals, particularly IL-1, implicated in paracrine senescence. (**Right**) The pro-tumor effects of cellular senescence. SnCs can dampen cytotoxic lymphocyte function by expressing certain surface molecules, including the non-canonical class I MHC molecule HLA-E and immune checkpoint PD-L1/2. The SASP also facilitates recruitment and differentiation of immunosuppressive cells, such as myeloid-derived suppressor cells (MDSCs) and M2-like macrophages, which inhibit NK and T cell function. Additionally, SnCs may contribute to tumor progression by promoting non-immunologic processes such as epithelial–mesenchymal transition (EMT), vasculogenesis, cancer cell reprogramming, malignant transformation, and hyperproliferation.

Senescence in preneoplastic cells may serve as a barrier to carcinogenesis while therapy-induced senescence in tumor cells may mediate the benefits of cancer therapies [232,233] (Figure 3). Along with the direct effects of SnCs and the SASP, a major contributor to the beneficial effects of SnCs may be to promote innate and adaptive immunity (Table 1).

### 4.1. Senescence as a Barrier to Tumorigenesis

Although Ras-activating mutations are observed in diverse cancers, oncogenic Ras induces senescence rather than proliferation when introduced into untransformed cells [49]. Thus, OIS may be a critical barrier limiting carcinogenesis. Studies with fibroblasts expressing H-Ras^G12V^ suggested that activation of p53 and induction of p21^CIP1^ and/or p16^INK4A^, resulting in senescence, may mediate the inability of oncogenic Ras on its own to transform primary cells [49]. Lymphocytes that escape N-Ras^G12D^-induced senescence show rapid proliferation and develop into aggressive but apoptosis-competent lymphomas in vivo [232]. Acute inactivation of the tumor suppressor PTEN in the prostate similarly leads to growth arrest and senescence in a p53-dependent manner, while concurrent inactivation of PTEN and p53 bypasses senescence and results in invasive cancer [234]. The SASP may serve a beneficial role in limiting progression from premalignancy [235], though resulting inflammation might ultimately have adverse consequences.

### 4.2. Senescence-Associated Growth Arrest in Cancer

Senescence may impact cancer treatment outcomes. In murine lymphoma models competent for p53 or INK4a, cyclophosphamide-induced senescence can enhance the response to chemotherapy [233]. In ovarian cancer, cisplatin resistance has been associated with impaired senescence initiation caused by increased expression of Aurora kinase A [236].

That SnCs can induce senescence in surrounding cells via paracrine or bystander senescence may further enhance their tumor-suppressive effects [11,237,238]. The SASP may be the primary driver of paracrine senescence [239,240]. NF-κB- and C/EBP-β-dependent SASP factors such as IL-8 and GRO create a self-amplifying secretory network by activating CXCR2, which enhances ROS and facilitates senescence induction. TGF-β promotes paracrine senescence through p16^INK4A^ and p21^CIP1^-dependent mechanisms, with TGFBR1 inhibitors partially mitigating this effect [237]. IGFBP7, secreted by BRAF^V600E^-driven SnCs, induces senescence and apoptosis in neighboring cells via MEK-ERK signaling and offers a potential mechanism for melanoma suppression [241]. Other soluble SASP components that have been identified to induce paracrine senescence include IL-1, VEGF, CCL2, and CCL20 [237]. Senescence may also be propagated by direct cell–cell transfer of ROS via gap junctions to trigger the DDR in neighboring cells [11].

### 4.3. Innate Immune Responses Activated by SnCs

A role for innate immunity in senescence surveillance was first established in studies of p53 reactivation in H-Ras^G12V^-induced liver cancer [27,28]. The resulting SnCs expressed inflammatory cytokines that recruit neutrophils and natural killer (NK) cells to eliminate SnCs but that can also target tumor cells, resulting in a potent tumor-suppressive effect. Senescence in melanoma cells induced by the Aurora kinase A inhibitor alisertib triggers NF-κB signaling that activates a SASP attracting neutrophils and macrophages [242]. Depleting macrophages in vivo weakens immune surveillance and diminishes tumor suppression [242]. The ability of the SASP to promote an M1 macrophage phenotype provides a mechanism by which SnCs may mediate their anti-tumor effects [243,244]. M0 macrophages exposed to extracellular vesicles released by SnCs elevated expression of M1-associated proinflammatory genes while suppressing M2 markers [245]. Modifying the SASP by suppressing autophagy may further augment M1-associated immune surveillance [244].

Along with phagocytes, NK cells serve important roles in senescence-associated immune surveillance [28]. Senescence disruption in B cell lymphoma reduces immune cell infiltration, particularly for NK cells, leading to increased therapy resistance and reduced survival rates [199]. Liver cancer cells undergoing H-Ras^G12V^-induced senescence promote NK cell activity via engaging the NK-activating receptor NKG2D and secreting NK cell-attracting chemokines such as CCL2, which amplifies NK-dependent tumor surveillance [246]. Treatment of myeloma cells with doxorubicin or melphalan induces SnCs with elevated surface expression of NK cell-engaging ligands including NKG2D ligands MICA/B and DNAM-1 ligand PVR to drive degranulation and tumor cell elimination [247,248,249]. That NK cell ligands can be upregulated by the DDR points toward a mechanism by which SnCs may enhance NK cell function [247,248].

The SASP also contributes to SnC stimulation of NK cell function. Media conditioned by SnCs enhance NK cell efficiency against both senescent and proliferating tumor cells [250]. Inducing senescence in K-Ras^G12D^/p53^null^ lung cancer models with MEK inhibitor trametinib or CDK4/6 inhibitor palbociclib upregulates immune-modulatory molecule ICAM-1 and the SASP component TNF-α via NF-κB activation, therefore facilitating NK cell-mediated tumor surveillance [251]. Like NK cell depletion, neutralizing TNF-α significantly compromises this anti-tumor response.

Epigenetic alterations also influence the immunogenicity of senescence through SASP modulation. The lack of BRD4, a key mediator of epigenetic transcription activation, results in reduced cytokine release and diminished NK cell activation by SnCs [119]. The polycomb-related complex 2 (PRC2) catalytic subunit EZH2, an epigenetic transcriptional repressor, suppresses the proinflammatory SASP and restrains SnCs’ ability to stimulate cytotoxic lymphocytes. Combining the EZH2 inhibitor tazemetostat with senescence-inducing agents such as trametinib and palbociclib significantly enhances immune infiltration and activates NK and T-cell-mediated tumor control in preclinical models of pancreatic ductal adenocarcinoma (PDAC) [252].

### 4.4. Activation of Adaptive Immune Responses by SnCs

Recent studies have confirmed that senescence can be a form of immunogenic cell stress [253] with the potential for TIS to activate adaptive immunity to further limit tumor progression and enhance therapeutic effects. Multiple features of SnCs have been proposed to drive T-cell-mediated cytotoxicity [254,255,256].

An initial recognition of the potential for T cells to be stimulated by SnCs came from studies of hepatocytes undergoing N-Ras^G12V^-driven senescence, where formation of SnCs induced CD4^+^ T cell-mediated tumor surveillance [29]. Radiation-induced senescence has been shown to facilitate NKT cell infiltration, providing a protective barrier against tumor development. Conversely, the failure of cells to undergo senescence due to deficiencies in IL-6 or Rb has been linked to accelerated osteosarcoma progression [257].

The SASP serves as a vital bridge between SnCs and adaptive immunity. Dexamethasone-induced senescence in lung adenocarcinoma cells leads to the release of chemokines such as CCL2, CCL4, CXCL1, and CXCL2, attracting T and NK cells to the tumor site [258]. SnCs induced by the Aurora kinase A inhibitor alisertib can secrete CCL5 through an NF-κB-dependent pathway, thereby contributing to T-cell-mediated tumor regression [259]. This anti-tumor effect is further enhanced when alisertib is combined with a T-cell-activating CD137 agonist antibody, providing synergistic benefits in melanoma models [259].

SnCs induced by either DNA damage agents or CDK4/6 inhibitors express antigen presentation machinery that potentiates T cell responses [260]. For example, SnCs induced by abemaciclib demonstrate increased surface levels of the β2M subunit of MHC I and present tumor antigens to the T cell receptor (TCR) of cytotoxic CD8^+^ T cells to elicit T-cell-dependent anti-tumor responses [261]. Combining abemaciclib with anti-PD-L1, an immune checkpoint inhibitor, results in further tumor suppression [261]. MHC II molecules are upregulated in oncogene-induced senescent melanocytes to facilitate T cell expansion [174]. Senescent acute myeloid leukemia (AML) cells triggered by cytosine arabinoside also display increased MHC expression to effectively activate T cell proliferation [262]. This can be enhanced by the anti-PD-1 antibody nivolumab [262]. These synergies suggest a potential combination strategy of senescence-inducing therapies with ICB to achieve enhanced cancer control.

**Table 1:** Immunostimulatory senescent cells in cancer.

Senescence Induction Methods	Cancer Type	Affected Immune Cell Population
H-Ras^G12V^ and p53 reactivation	Liver cancer	PMN [27]
Alisertib	Melanoma	PMN and macrophages [242]
Carbon tetrachloride	Hepatocellular carcinoma	M1 macrophage [243]
Temozolomide	Glioblastoma	Increased M1 macrophages and decreased MDSCs [244]
IR	Lung cancer	M1 macrophages [245]
Cyclophosphamide	B cell lymphoma	NK cells [199]
H-Ras^G12V^	Liver cancer	NK cells [246]
Chemo agents	multiple myeloma	NK cells [247]
Doxorubicin	multiple myeloma	NK cells [248]
Trametinib + Palbociclib	KP lung cancer	NK cells [251]
N-Ras^G12D^	Liver cancer	NK cells [119]
Trametinib + Palbociclib + Tazemetostat	PDAC (KPC model)	NK and T cells [252]
N-Ras^G12V^	Liver cancer	CD4^+^ T cells [29]
IR	Osteosarcomas	NKT cells [257]
Alisertib	Melanoma	CD8^+^ T cells [259]
Dexamethasone	Lung adenocarcinoma	NK and T cells [258]
Abemaciclib	Mammary carcinoma	T cells [261]
N-Ras^Q61K^ or B-Raf^V600E^	Primary melanocytes	T cells [174]
Cytarabine or Palbociclib	AML	T cells [262]
Doxorubicin	Melanoma	DCs and T cells [254]
IR + veliparib	Multiple cancers	DCs, NK, and T cells [26,256]
N-Ras^G12D^	Liver cancer	Increased CD8^+^ T cells and decreased MDSCs [255]
Doxorubicin	Metastatic breast cancer	CD8^+^ T cell [263]
Trametinib + Palbociclib	PDAC (KPC model)	CD8^+^ T cells [264]
Abemaciclib	Melanoma	T cells [265]
AZD1152	Melanoma	T cells [266]
Irinotecan + Cisplatin	Ovarian cancer	DCs and T cells [267]
IFN-γ	Multiple cancers	T cells [268]

IR: ionizing radiation; PDAC: pancreatic ductal adenocarcinoma; AML: acute myeloid leukemia; PMN: polymorphonuclear leukocytes; MDSC: myeloid-derived suppressor cell; NK: natural killer cell; DCs: dendritic cells; IFN: interferon.

### 4.5. Senescence as a Strategy to Overcome an Immunosuppressive Tumor Microenvironment

To leverage the immunostimulatory properties of senescence and the SASP, recent studies have re-examined senescence induction in tumor cells to reshape the microenvironment from immune evasion toward immune recognition. Reexamining the OIS model of N-Ras^G12D^-induced senescence in liver cancer revealed a shift to increased lymphocytes and depletion of Gr1^+^ myeloid-derived suppressor cells (MDSCs) [255]. These SnCs display enhanced responses to IFNγ, potentiating antigen processing and presentation that support cytotoxic T cell activity and tumor suppression [255]. Analogously, doxorubicin-induced senescence modifies the immune landscape by promoting CD8^+^ T cell recruitment, while clearing SnCs by senolysis reverses this infiltration [263].

The potential for TIS to overcome immunosuppression in the tumor microenvironment presents a promising strategy to enhance response to immunotherapies (Table 2). Inducing senescence with doxorubicin in brain metastases from mammary carcinoma enhances the efficacy of anti-PD-1 immune checkpoint blockade, which suggests a synergistic approach of combining immunotherapy with senescence induction [263]. In PDAC models, combining trametinib and palbociclib to induce senescence leads to the secretion of NF-kB-dependent pro-angiogenic SASP factors such as VEGF, PDGFA/B, FGF2, and MMPs, which promote vascular remodeling and enhance the delivery and efficacy of genotoxic agents like gemcitabine. Importantly, this senescence-associated vascular remodeling also facilitates T cell migration into the tumor, thereby priming immunologically cold tumors for increased responses to anti-PD-1 [264]. Melanoma resistance to ICB has been linked to T cell exclusion mediated by inhibited antigen processing/presentation, reduced IFN-γ signaling, and compromised immune modulation. Treatment with abemaciclib disrupts this mechanism by leveraging the SASP to enhance T cell infiltration, which sensitizes melanomas to therapies like anti-PD-1 and anti-CTLA-4 [265]. Downregulating p21^CIP^ expression in melanoma cells decreases the senescence triggered by the Aurora kinase B inhibitor AZD1152 and limits AZD1152-induced T cell cytotoxicity [266]. In ICB-resistant ovarian cancer, utilizing SnCs as a form of cell therapy significantly improves the efficacy of anti-PD-1 treatments by stimulating CD8^+^ T cell and dendritic cell (DC) activation [267]. Providing a potential positive feedback loop, IFNγ generated by T cells activated by ICBs can promote tumor cell senescence via Stat1 activation and p21^CIP^ induction. Tumors lacking Stat1 or p21^CIP^ fail to undergo senescence and display resistance to anti-PD-L1 and anti-LAG3 [268]. Taken together, cellular senescence may have an underappreciated role in immunotherapy responses.

### 4.6. Immunogenic Senescent Tumor Cells Function as Cancer Vaccines

Building on observations suggesting that SnCs formed in tumors during genotoxic therapy might contribute to subsequent anti-tumor immune response, a 2012 study directly tested vaccination with senescent tumor cells [26]. SnCs induced by combining ionizing radiation and the PARP inhibitor veliparib displayed preventive and therapeutic effects against tumor growth in multiple models, associated with an immunogenic SASP and mediated through a CD8^+^ T-cell-dependent mechanism [26]. Subsequent research from our lab and others has explored the potential of inducing immunogenic senescence to form cancer vaccines. While our studies focused on radiation and veliparib-induced senescence, others have found similar vaccine-like properties in doxorubicin-induced senescent melanoma cells and irinotecan–cisplatin-induced senescent ovarian cancer cells [254,256,267]. Due to their enhanced antigenicity and adjuvanticity, immunogenic SnCs recruit and activate DCs, the indispensable players supporting full T cell activation and immune surveillance against cancer [254,256,267].

Notably, it is specifically viable SnCs, rather than lysed, apoptotic, or stressed cells, that trigger effective CD8^+^ T-cell-driven anti-tumor immune responses [26,254,256]. Via their vaccine effects, immunogenic SnCs can suppress both primary tumors and metastases on their own, but also synergize with other cancer therapies, including irradiation and ICB [26,254,256,267]. SnCs lacking STING fail to activate DCs effectively and display a diminished vaccine effect [256]. Non-senescent cells treated with the STING agonist DMXAA also fail to similarly enhance DC activation or promote anti-tumor immunity [256,267], underscoring the importance of multiple features of SnCs that contribute to an effective immune response. While immunogenic SnCs may not be practical as a therapy, a broader implication of these findings is that TIS might be leveraged as a vaccine in situ, potentiating an anti-tumor immune response after genotoxic or targeted therapy.

## 5. Senescence as an Enemy in Cancer Treatment

Under normal physiological conditions, timely removal of SnCs is important for tissue health and overall organismal homeostasis, while their accumulation can contribute to inflammation and age-related disease. Although cellular senescence is an important tumor suppressive mechanism that limits the proliferation of damaged cells, it can paradoxically become an adversary in cancer treatment. The persistence of inflammatory SnCs within the TME may promote tumorigenesis, tumor growth, and immunosuppression, antagonizing cancer therapies [18,269]. The detrimental effects of senescence have been linked to therapy resistance, metastasis, relapse, and toxicities (Figure 3). Strategies that modulate the SASP or eliminate inflammatory SnCs are being explored as avenues for cancer therapy improvement.

### 5.1. Impacts of Senescence on Malignant Transformation and Tumor Growth

Cellular senescence and associated inflammation linked to aging and stress have long been implicated in promoting malignancy. Fibroblasts rendered senescent by proliferation or by oncogenic or genotoxic stress have been implicated as drivers of cancer initiation, progression, metastasis, and therapy resistance. Co-injecting pre-malignant or malignant epithelial cells with senescent fibroblasts significantly increased tumor formation and growth rates, suggesting that SnCs provide a permissive niche [270]. Non-malignant epithelial cells exposed to senescent fibroblasts display changes indicative of invasive behavior and early malignant transformation, such as increased migration and nuclear atypia [271,272]. Reduced tumor formation upon eliminating SnCs by targeting p16^INK4A^-positive cells in the transgenic INK-ATTAC mouse model may reflect depletion of this permissive niche [96]. This effect is observed irrespective of the initial senescence trigger, reinforcing the broad nature of SnC-related growth promotion [270,273].

Effects of SnC conditioned medium on non-senescent cells [271,272] suggest a role for inflammatory mediators in the SASP in promoting malignancy. Inhibiting the cGAS-dependent SASP in senescent fibroblasts diminishes their effects [273]. Multiple mediators and target pathways have been implicated. Senescent mesenchymal cells release IL-6 that drives Stat3 signals to enhance breast cancer cell proliferation and migration [274]. Release of amphiregulin (AREG) increases cancer cell proliferation and survival via activation of the epidermal growth factor receptor (EGFR) pathway in nearby cells [275]. Other SASP factors, such as osteopontin and matrix MMPs, have also been implicated in promoting cell proliferation and cancer progression [271,276,277].

**Table 2:** Therapy-induced senescence potentiates immunotherapy.

Senescence Induction Methods	Cancer Type	Immunotherapy Agent
Mitoxantrone	Prostate cancer	α-PD-1/PD-L1 Ab [275]
Doxorubicin	Melanoma	α-PD-L2 Ab [37]
Alisertib	Melanoma	α-CD137 Ab [259]
Abemaciclib	Mammary carcinoma	α-PD-L1 Ab [261]
IR + Veliparib	Multiple cancers	α-PD-L1 Ab [256]
Doxorubicin	Metastatic breast cancer	α-PD-1 Ab [263]
Trametinib + Palbociclib	PDAC (KPC model)	α-PD-1 Ab [264]
Abemaciclib	Melanoma	α-CTLA4 Ab [265]
AZD1152	Melanoma	α-CTLA4 Ab [266]
Irinotecan + Cisplatin	Ovarian cancer	α-PD-1 Ab [267]

IR: ionizing radiation; PDAC: pancreatic ductal adenocarcinoma; α: anti; Ab: antibody; PD-1: programmed cell death protein 1; PD-L1: programmed death-ligand 1; CTLA4: cytotoxic T-lymphocyte associated protein 4.

### 5.2. Senescent Cell Effects on Invasion and Metastasis

SnCs can contribute to metastasis both by promoting the migration and extravasation of cancer cells from tumors as well as by forming a pre-metastatic niche that supports invasion and colonization in distant tissues. As an example, breast cancer cells implanted with HER2-induced SnCs exhibit a marked increase in metastatic capability via non-cell autonomous mechanisms [278]. This enhancement of metastatic potential is also observed following chemotherapy, where TIS in stromal cells and fibroblasts creates a nurturing environment conducive to tumor colonization in distant organs such as the liver, lungs, and bones [279].

The pro-metastatic effect of senescence is largely attributed to SASP released by senescent stroma and/or tumor cells. Conditioned medium from senescent fibroblasts induced by bleomycin alters breast cancer cell morphology and migration and advances them to an aggressive state [280]. Senescent colon cancer cells treated with fluorouracil also display a SASP that induces epithelial-to-mesenchymal transition (EMT) and enhances tumor cell invasion [281]. Specific SASP factors may play predominant roles in these pro-metastatic effects. MMP-1 and MMP-2 produced by senescent fibroblasts can promote skin carcinoma cell migration and keratinocytes EMT [282]. CXCL12 released by BRAF^V600E^-induced senescent thyrocytes directs thyroid carcinoma cell invasion [283]. IL-6 secreted by senescent osteoblasts contributes to increased osteoclast genesis and bone metastases [284]. Chemerin, a newly identified SASP factor, boosts the migration of cutaneous squamous cell carcinoma cells by activating the MAPK signaling pathway [285].

SnCs influence vascular remodeling that can further facilitate tumor metastasis. Co-transplantation of senescent thyrocytes with thyroid cancer cells has been shown to promote the development of lymphatic vessel networks and metastatic foci in lymph nodes [283]. Senescent melanoma cells secrete SFRP2, a Wnt antagonist that stimulates angiogenesis and accelerates melanoma metastasis [286]. Other SASP factors implicated in angiogenesis include vascular endothelial growth factor (VEGF) and connective tissue growth factor (CTGF) [287,288]. Suppressing the SASP with anti-inflammatory agents such as metformin can reduce pathological neovascularization driven by senescence, offering a potential strategy for mitigating cancer metastasis [289].

### 5.3. Stimulation of Therapy Resistance via Promoting Survival, Dormancy, EMT, and Stemness

The accumulation of both stromal and cancer SnCs within tumors during therapy is likely a significant mechanism underlying resistance. Doxorubicin-induced senescent endothelial cells in the thymus contribute to a chemo-resistant microenvironment. This supports the survival and eventual relapse of residual cancer cells through the release of SASP factors such as IL-6 and Timp-1 [290]. IL-6 secreted by senescent endothelial cells via PI3K/AKT signaling pathways appears to be sufficient to confer chemoprotective effects [291]. In breast cancer, p53-dependent senescence shields cancer cells from apoptosis via SASP and consequently reduces the efficacy of doxorubicin [292]. The SASP released by Ras-induced senescent mesothelioma cells activates Stat3 signaling in neighboring cells to foster the development of an EMT-like, clonogenic, and chemo-resistant subpopulation of cancer cells [293].

Senescence may also contribute to stemness that furthers tumor recurrence and therapy resistance. Ectopic expression of Yamanaka factors (OCT4, SOX2, KLF4, and cMYC) in mice paradoxically leads to both cell reprogramming and senescence [294]. Key components of the senescence machinery, including p16^INK4A^, p21^CIP^, p53, and H3K9me3, have complementary roles as stemness regulators [295]. Inhibiting SASP factors such as IL-6 can reduce cell de-differentiation, implying that SnCs may enhance cellular plasticity via paracrine effects [296]. RAS^G12V^-induced SnCs can secrete a Stat3-dependent SASP that leads to the development of a subpopulation of progenitor-like cancer cells exhibiting resistance to chemotherapy agents like pemetrexed [293]. Doxorubicin-induced senescence in liver cancer increases the expression of genes associated with reprogramming and liver stemness, including c-MYC and EpCAM, as well as the multidrug resistance gene ABCG2 [297]. This enables adjacent tumor cells to upregulate tumor-initiating capabilities [297]. Exposing breast cancer MCF-7 cells to a conditioned medium from SnCs or to SASP factors like IL-6 and IL-8 induces stemness characteristics such as elevated CD44 expression and self-renewal capabilities [298]. The senescence-mediated promotion of tumor cell reprogramming extends to hematological malignancies as well. DNA damage-induced senescent myeloma cells facilitate the emergence, sustenance, and migration of stem-like cancer cells through a CHK2-dependent SASP [299]. Evidence also suggests senescence gives rise to cancer stemness in B-cell lymphoma, B-cell chronic lymphocytic leukemia (B-CLL), and AML [295].

### 5.4. Senescence-Induced Immunosuppression and Strategies for Improved Cancer Treatment

Cellular senescence has been implicated in dampening immune responses (Figure 3 and Table 3) [300,301]. Systemic IR-induced senescence compromises the phagocytic capabilities of DCs and macrophages, which diminishes the proliferation of splenic B and T cells. Studies have demonstrated that genetic elimination of p16^INK4^-expressing SnCs in the p16-3MR model restores antigen-presenting cell (APC) function and rejuvenates both T and B cell populations [302]. The removal of p16^INK4^-high senescent malignant cells boosts T cell infiltration and reduces tumor-promoting macrophages in glioblastoma, thereby aiding in tumor suppression and enhancing survival rates in tumor-bearing mice [303].

The SASP can contribute to the immunosuppressive effects of SnCs. In prostate tumors lacking PTEN and treated with docetaxel, SnCs exhibit sustained Jak2/Stat3 activation that induces an immunosuppressive SASP, leading to suppressive myeloid cell accumulation while depleting CD4^+^, CD8^+^, and NK cells. Genetically or pharmacologically inhibiting Jak2/Stat3 triggers a robust anti-tumor immune response and re-sensitizes tumors to docetaxel [304]. In fact, the accumulation of MDSCs has long been implicated in the decline of T cell cytotoxicity and increased tumor susceptibility in aged mice [305]. Conditioned medium from p27^Kip^-driven senescent fibroblasts encourages bone marrow stem cell differentiation into CD11b^+^Ly6G^hi^ MDSCs that suppress cytotoxic immunity and foster a pro-tumorigenic environment. Neutralizing IL-6 from these fibroblasts reduces MDSC levels, reactivates T cell function, and delays tumor progression [306]. Injection of palbociclib-induced senescent fibroblasts also leads to MDSC infiltration through NF-kB pathways and accelerates melanoma growth in immunocompetent hosts [307]. Interestingly, tumor-infiltrating MDSCs can antagonize senescence by secreting IL-1RA to block IL-1α-mediated OIS in neighboring cells and further decrease overall cancer treatment efficacy. CCR2 antagonist treatment greatly reduces Gr-1^+^ myeloid cell infiltration, thus improving chemotherapy in PTEN-null prostate cancer [308]. In hepatocellular carcinoma, CCL2 secreted by N-Ras^G12V^-induced senescent hepatocytes disrupts the maturation of CCR2^+^ myeloid precursors. The resulting immature myeloid cells efficiently suppress NK cell activity and significantly facilitate tumor progression [309].

Notch signaling has also emerged as a key regulator of the immunosuppressive functions of SnCs. Elevated Notch signaling in N-Ras^G12V^-induced senescent hepatocytes leads to a TGF-β-enriched SASP that dampens T-cell-mediated immune surveillance [178]. Radiation-induced senescence in the lung fosters a pro-tumorigenic microenvironment characterized by increased neutrophil infiltration and amplified Notch signaling. Notch inhibition, however, can markedly reduce radiation-enhanced metastases [310]. Under hypoxic conditions, TGF-β can trigger senescence by repressing E2F targets and inducing a SASP that creates an immunosuppressive environment, which results in elevated myeloid cells and diminished immunotherapy success [311]. Knockout of TGF-β receptors in lung cancer cells alleviates the senescence phenotype and restores immune balance [311]. In colorectal cancer, senescent tumor cells driven by oxidative stress upregulate CXCL12 and CSF1 secretion. Increased levels of CXCL12 lead to reduced CXCR4 expression on CD8^+^ T cells and inhibit their chemotactic migration. CSF1 promotes M2 macrophage differentiation that further impedes CD8^+^ T cell activation. CXCL12/CSF1 neutralization therefore enhances T cell infiltration and the efficacy of anti-PD-1 treatment [312]. Another immunosuppressive SASP factor is Galectin-9, which is secreted by alkylating agent-induced senescent melanoma cells through mitofusin 1 (Mfn1). Silencing Mfn1 in melanoma promotes tumor immune infiltration and enhances chemotherapy efficiency [313].

Metabolites secreted by SnCs can also contribute to immune suppression. Senescent hepatic stellate cells (HSCs) can upregulate prostaglandin production via TLR2 signaling and the increase in PGE2 promotes regulatory T cells (Tregs) in the liver and diminishes cytotoxic T cell activity. Blocking the PGE2 receptor PTGER4 rejuvenates the anti-tumor immunity by elevating CD103^+^ DCs and CD8^+^ T cells while reducing Tregs, thus protecting against high-fat-diet-induced hepatocellular carcinoma [314].

Senescent dermal fibroblasts can display increased surface expression of the non-classical MHC molecule HLA-E that contributes to immunosuppressive effects by activating the NKG2A-mediated inhibitory pathway in NK and CD8^+^ T cells [175]. The induction of HLA-E may be amplified by IL-6 in both a cell-autonomous and non-autonomous manner. Blocking the interaction between HLA-E and NKG2A enhances immune surveillance of SnCs in vitro, offering a strategy to alleviate senescence-associated immunosuppression [175]. SnCs can also upregulate the immune checkpoint molecule PD-L1, leading to CD8^+^ T cell inhibition [34,36]. The mechanisms behind PD-L1’s upregulation include enhanced E2F1 and/or NF-kB-mediated transcription, reduced proteasome activity, and cGAS-STING-driven paracrine effects [33,34,36]. Anti-PD-1 treatments may enhance immune surveillance of SnCs and reduce age-related dysfunction in mice [36]. Combining senescence-inducing chemotherapy, such as mitoxantrone, with anti-PD-1/PD-L1 therapies leads to tumor regression via cytotoxic T cell activation [275]. SnCs induced by doxorubicin or the CDK4/6 inhibitor palbociclib also upregulate PD-L2, another immune checkpoint receptor ligand that disrupts T cell function through PD-1 engagement and facilitates MDSC recruitment [37]. Targeting this immunosuppressive mechanism with PD-L2 blockade in combination with chemotherapy results in a significant tumor reduction in murine syngeneic models [37]. The so-called “Don’t Eat Me” signals CD47 and CD24 are also increased in TIS, suppressing macrophage phagocytosis and efferocytosis. Blocking CD47-mediated SIRPα signaling with antibodies or FAB fragments partially reverses SnC-mediated macrophage suppression [176]. These findings converge on the potential for immunotherapies to overcome SnC-induced immune suppression.

**Table 3:** Immunosuppressive senescent cells in cancer.

Senescence Induction Methods	Cancer Type	Affected Immune Cell Population
Docetaxel	PTEN loss prostate cancer	Increase Gr1^+^ MDSCs but decrease T and NK cells [304]
p27^Kip1^	Squamous cell carcinoma	Increase CD11b^+^Ly6G^Hi^ MDSCs and Tregs [306]
Palbociclib	Melanoma	Promote the recruitment of Gr1^+^ MDCS [307]
Pten-loss	PTEN loss prostate cancer	Increase MDSCs [308]
N-Ras^G12V^	Liver cancer	Increase MDSCs [309]
N-Ras^G12V^	Liver cancer	Reduce CD3^+^ T cells [178]
IR	Lung metastases	Promote Ly6G^+^ neutrophil recruitment [310]
TGF-β	Lung cancer	Increase infiltration of immune-suppressive cell types [311]
ROS	Colorectal Cancer	Enhance M2 macrophage polarization [312]
Temozolomide	Melanoma	Suppress tumor immune infiltrates [313]
Metabolites (DCA and LTA)	Hepatocellular carcinoma	Suppress CD8^+^ T cells [314]
Mitoxantrone	Prostate cancer	Promote PD-L1 expression in tumors [275]
Doxorubicin	Melanoma	PD-L2^+^ senescent cells dampen T cell activity and promote CD11b^+^Gr1^+^ MDSC recruitment [37]
H-Ras^G12V^	Glioblastoma	Decrease T cells and increase tumor-promoting macrophages [303]
Palbociclib	Pancreatic carcinoma	Inhibit macrophage phagocytosis and efferocytosis [176]

ROS: reactive oxygen species; DCA: dichloroacetic acid; LTA: lipoteichoic acid; NK cells: natural killer cells; MDSCs: myeloid-derived suppressor cells; Tregs: regulatory T cells.

### 5.5. Lifestyle Interventions to Influence Metabolism and Modulate Senescence

Dietary interventions have long been recognized to extend lifespan and mitigate oxidative stress [315]. They can also influence the induction and characteristics of cellular senescence [316]. For example, short-term caloric restriction can reduce markers of cellular senescence and the SASP in both murine models and middle-aged humans, potentially slowing aging-related phenotype progression [317]. Dietary methionine restriction may also delay senescence and reduce inflammatory SASP factors by enhancing the production of hydrogen sulfide (H_2_S), an endogenous antioxidant, through the transsulfuration pathway [318,319]. Omega-3 fatty acids, particularly eicosapentaenoic acid (EPA) and docosahexaenoic acid (DHA) found in fish oil supplements, have been shown to decrease H_2_O_2_-induced DNA double-strand breaks, lipid peroxidation, and resulting senescence by promoting the expression of NRF2 and subsequent antioxidant proteins [320]. Aerobic exercise has also been found to mitigate senescence and inflammation to protect naturally aged mice from cancer development [321].

### 5.6. “One-Two Punch” Therapies Using Senolytics against Cancer

The elimination of p16^high^ SnCs following doxorubicin chemotherapy can enhance breast cancer treatment response, reduce the likelihood of metastasis/relapse, and diminish chemotherapy-associated toxicity [269]. This approach suggests a potential pharmacological method to mitigate the negative impact of senescence by targeting and eliminating SnCs with senolytics (Table 4). Senolytics have shown considerable promise in addressing age-related diseases by eliminating naturally formed SnCs [322,323]. In the context of cancer, they might be applied as a “one-two-punch” strategy where therapy that may produce TIS within the tumor is followed by senolytic treatment to target the vulnerabilities of SnCs [23,24].

These and other considerations have stimulated ongoing efforts to identify safe and selective senolytics (as reviewed by [324]). It has long been appreciated that changes in the expression or activity of anti- and pro-apoptotic Bcl-2 family proteins may mediate the persistence of SnCs [325,326]. Targeting Bcl-xL with siRNA was shown to selectively eliminate SnCs while leaving proliferating or quiescent cells unaffected [130]. BH3 mimetics such as navitoclax (ABT-263), which inhibits Bcl-xL, Bcl-2, and Bcl-w, can trigger apoptosis in SnCs in vitro and in vivo, leading to the resolution of multiple features of aging [95,327,328,329]. Treatment with ABT-737, another multitargeted Bcl-2 family inhibitor, clears K-Ras^G12D^-induced SnCs from the pancreas and reduces the formation and progression of premalignant lesions that lead to PDAC [330]. Applying navitoclax to eliminate senescent cells induced by doxorubicin or etoposide enhances tumor suppression in both immunodeficient and immunocompetent models [331,332]. Combination treatment of navitoclax with olaparib displays synergistic effects on ovarian xenografts [333]. Toward reducing off-target toxicity, a galacto-conjugate of navitoclax, Nav-Gal, is activated specifically within SnCs upon SA-β-Gal-mediated cleavage [334]. Nav-Gal can delay tumor progression when combined with cisplatin [334]. Nav-Gal also parallels navitoclax in its ability to inhibit lung metastases in a mouse TNBC model via elimination of senescent lung endothelial cells induced by palbociclib [335].

Bcl-xL-specific inhibitors such as A1331852 or A1155463 and degraders based on proteolysis-targeting chimera (PROTAC) technology display potency in eliminating senescent cancer cells [336,337], but the Bcl-2-specific inhibitor ABT-199 shows inconsistent senolytic activity across various models [328,329,332,333,338,339]. The Mcl-1 inhibitor S63845 is another potential senolytic [332,340]. The sequential application of docetaxel followed by S63845 effectively eliminates SnCs and suppresses the growth of prostate cancer. This combination treatment also revitalizes anti-tumor immunity by diminishing immunosuppressive cells and amplifying cytotoxic immune markers [340]. Bcl-2 family proteins are likely to serve as indirect targets of other senolytics. The HDAC inhibitor panobinostat appears to mediate its effects via downregulating Bcl-xL expression [341,342].

Another strategy to target the apoptotic threshold is exemplified by the best-studied senolytic agent to date, a combination of dasatinib and quercetin (D + Q) [130,343]. D + Q has shown potential in preclinical models for controlling diverse age-associated conditions including pulmonary fibrosis and neurodegeneration [344,345] along with promising activity in clinical trials. A range of other flavonoids and derivatives such as fisetin and GL-V9 display senolytic activity [140,333,336,346]. Senolytic flavonoids may indirectly antagonize anti-apoptotic Bcl-2 family proteins via elevating ROS levels and downstream signaling [346]. A caveat is that D + Q may be less effective in eliminating TIS than replicative senescent cells [347].

Beyond the Bcl-2 family proteins, a wide range of agents targeting other pathways have been proposed as senolytics. Compounds targeting BRD4 such as the inhibitor JQ1 and degrader ARV825 have emerged as potent senolytics by promoting autophagy-induced cell death. Combining ARV825 with doxorubicin improves therapy outcomes in liver cancer [348]. The mTOR inhibitor AZD8055 eradicates SnCs induced by the CDC7 inhibitor XL413, which may suggest an alternative combination treatment for liver cancer [341]. Cardiac glycosides, such as digoxin, digitoxin, and ouabain, may be another class of senolytics [349,350]. SnCs exhibit increased susceptibility to the ferroptosis inducer RSL3 compared to non-SnCs, likely due to elevated levels of cytosolic lipid peroxidation [351]. Canagliflozin, an inhibitor of sodium–glucose co-transporter 2 (SGLT2), may indirectly target senescence caused by a high-fat diet in mouse adipose tissue through activating AMPK signaling and potentiating immune surveillance [352]. These studies have expanded the spectrum of agents for one-two-punch therapy.

Beyond small molecules, strategies for senolysis have extended to peptides, antibodies, vaccines, and cell-based therapies targeting senescence-associated features [353]. FOXO4-binding peptide E2 disrupts the interaction between FOXO4 and TP53, leading to the reactivation of TP53 transcription that induces apoptosis in SnCs [354]. Antibody-dependent cytotoxicity and antibody–drug conjugates that target senescence-specific surface antigens have been demonstrated [180,355]. Senolytic vaccination with an SnC-specific marker cleared marker-positive cells [356,357]. Senolytic CAR T cells targeting an SnC surface marker, uPAR, extended mouse survival in lung adenocarcinoma models treated with a senescence-inducing regimen of palbociclib and trametinib [21,358]. Senolytic CAR-T cells have since been reported targeting other SnC markers [359,360]. These and related advances summarize the dynamic and evolving landscape of senolytic therapies that aim to boost cancer treatment strategies with improved outcomes and reduced toxicity.

**Table 4:** Senolytics in cancer.

MOA	Senolytic Agent	Senescence Induction Methods	Cancer Type
Tyrosine kinase inhibitor + flavonoid derivative	Dasatinib and quercetin	Doxorubicin	Liver Cancer [347]
Flavonoid derivative	Fisetin	Olaparib	Ovarian cancer [333]
	GL-V9	Doxorubicin	Breast cancer [346]
Bcl-2/Bcl-xL/Bcl-w inhibitor	ABT263	Doxorubicin or etoposide	Lung and breast cancer [331]
	ABT263	SMARCB1 inhibition	Multiple cancer cell lines [329]
	ABT263	Olaparib	Ovarian [333]
	ABT263	Doxorubicin	Breast cancer [332]
	ABT263	Radiation and temozolomide	Glioblastoma [328]
	ABT737	K-Ras^G12V^	Pancreatic cancer [330]
Selective Bcl-xL inhibitor	A1331852, A1155463	Radiation and temozolomide	Glioblastoma cell lines [328]
Selective Bcl-2 inhibitor	ABT199	IR	Sarcoma cell lines [338]
	ABT199	Palbociclib + fulvestrant	Breast cancer [339]
MCL-1 inhibitor	S63845	Doxorubicin or etoposide	Breast cancer [332]
	S63845	Docetaxel	Prostate cancer [340]
Galacto-conjugation of ABT263	Nav-Gal	Cisplatin	A549 xenograft [334]
	Nav-Gal	Palbociclib	Breast cancer lung metastasis
BET degrader	ARV825	High-fat diet or doxorubicin	Liver cancer [348]
Cardiac glycosides	Digoxin Digitoxin Proscillaridin A Ouabain	Therapy induced senescence	Multiple cancer types [349,350]
mTOR inhibitor	AZD8055	CDC7 inhibitor XL413	Hepatocellular carcinoma [341]
HDAC inhibitor	Panobinostat Decrease Bcl-xl	Taxol, cisplatin	NSCLC and HNSCC [342]
Senolytic peptide	FOXO4-binding peptide ES2	BRAF inhibitor Dabrafenib	Melanoma [354]
Senolytic CAR-T	uPAR-specific CAR-T cells	Trametinib + Palbociclib	Lung cancer [21]

HDAC: histone deacetylase; CAR-T: chimeric antigen receptor T-cell; NSCLC: non-small cell lung cancer; HNSCC: head and neck squamous cell carcinoma; uPAR: urokinase-type plasminogen.

## 6. Conclusions

SnCs can display a diverse range of effects on cancer progression attributed to differences in senescence triggers, genetic backgrounds, tissue-specific contexts, and stages of senescence. This diversity emphasizes the significant heterogeneity and dynamic nature of SnCs, meriting the discovery of novel cellular markers that reflect the senescence stage and functional implications. A deeper analysis of the balance between the beneficial and detrimental effects of senescence on cancer therapy is needed to refine treatment strategies that specifically modulate SnC formation and/or functions. One goal of ongoing research is to minimize the negative impacts of SnCs while enhancing their positive therapeutic effects.

Recent technological advancements, including single-cell/nucleus RNA sequencing, have revolutionized our understanding of the complex regulatory mechanisms behind SnC heterogeneity [361,362,363,364]. These technologies provide insights into gene signatures, cell–cell interactions, and potential therapeutic targets. Spatial transcriptomics is an emerging tool that surveys gene expression at cellular detail within tissue, enabling a comprehensive analysis of the tumor microenvironment (TME) [365]. This technique may be particularly valuable for identifying SnCs in tumors and characterizing their impacts on surrounding cancer cells and immune and non-immune stroma to dissect juxtacrine and paracrine signaling in the TME.

The SASP is likely the major determinant of autocrine and paracrine effects of cellular senescence. Toward addressing the complexity of the SASP, advancements in proteomics and antibody array technologies have improved SASP component profiling [361]. The development of chemical biology tools for precise protein labeling and enrichment, such as APEX and TurboID [366,367], creates new opportunities for investigations into SASP characteristics. Future studies are needed to understand regulation of the SASP in relation to immune responses and cancer progression, as well as to identify modulators that can redirect the SASP to promote immunogenicity and reduce inflammatory signals.

Although TIS has the potential to drive cancer resistance and recurrence, pro-senescent therapies might also encourage immunogenic senescence as an in situ vaccine to boost anti-tumor immune responses and enhance cancer treatment outcomes. A concern is that strategies to promote immunogenic senescence in the tumor will also increase normal tissue senescence, leading to long-term toxicities including inflammation, fibrosis, organ dysfunction, and frailty [368]. In turn, the risk that SnCs formed in the tumor may re-enter a proliferative state and potentially drive recurrence [67,369] further argues that following treatment, any persistent SnCs may need to be eliminated, as with senolytics. Alternatively, forming immunogenic SnCs from patient tumor cells ex vivo in order to activate autologous immune cells such as DCs or T cells in vitro may provide a practical route to cell-based vaccines and adoptive cell therapies that can safely leverage immunogenic senescence for personalized immunotherapy.
